# Rapid and effective oxidative pretreatment of woody biomass at mild reaction conditions and low oxidant loadings

**DOI:** 10.1186/1754-6834-6-119

**Published:** 2013-08-26

**Authors:** Zhenglun Li, Charles H Chen, Eric L Hegg, David B Hodge

**Affiliations:** 1 Department of Chemical Engineering and Materials Science, Michigan State University, East Lansing, USA; 2 DOE Great Lakes Bioenergy Research Center, Michigan State University, East Lansing, USA; 3 Department of Biochemistry & Molecular Biology, Michigan State University, East Lansing, USA; 4 Department of Biosystems & Agricultural Engineering, Michigan State University, East Lansing, USA; 5 Department of Civil, Environmental and Natural Resources Engineering, Luleå University of Technology, Luleå, Sweden

**Keywords:** Biofuels, Bioenergy, Chemical pretreatment, Hybrid poplar, Copper, Cu(bpy), Lignin, Oxidation, Hydrogen peroxide

## Abstract

**Background:**

One route for producing cellulosic biofuels is by the fermentation of lignocellulose-derived sugars generated from a pretreatment that can be effectively coupled with an enzymatic hydrolysis of the plant cell wall. While woody biomass exhibits a number of positive agronomic and logistical attributes, these feedstocks are significantly more recalcitrant to chemical pretreatments than herbaceous feedstocks, requiring higher chemical and energy inputs to achieve high sugar yields from enzymatic hydrolysis. We previously discovered that alkaline hydrogen peroxide (AHP) pretreatment catalyzed by copper(II) 2,2΄-bipyridine complexes significantly improves subsequent enzymatic glucose and xylose release from hybrid poplar heartwood and sapwood relative to uncatalyzed AHP pretreatment at modest reaction conditions (room temperature and atmospheric pressure). In the present work, the reaction conditions for this catalyzed AHP pretreatment were investigated in more detail with the aim of better characterizing the relationship between pretreatment conditions and subsequent enzymatic sugar release.

**Results:**

We found that for a wide range of pretreatment conditions, the catalyzed pretreatment resulted in significantly higher glucose and xylose enzymatic hydrolysis yields (as high as 80% for both glucose and xylose) relative to uncatalyzed pretreatment (up to 40% for glucose and 50% for xylose). We identified that the extent of improvement in glucan and xylan yield using this catalyzed pretreatment approach was a function of pretreatment conditions that included H_2_O_2_ loading on biomass, catalyst concentration, solids concentration, and pretreatment duration. Based on these results, several important improvements in pretreatment and hydrolysis conditions were identified that may have a positive economic impact for a process employing a catalyzed oxidative pretreatment. These improvements include identifying that: (1) substantially lower H_2_O_2_ loadings can be used that may result in up to a 50-65% decrease in H_2_O_2_ application (from 100 mg H_2_O_2_/g biomass to 35–50 mg/g) with only minor losses in glucose and xylose yield, (2) a 60% decrease in the catalyst concentration from 5.0 mM to 2.0 mM (corresponding to a catalyst loading of 25 μmol/g biomass to 10 μmol/g biomass) can be achieved without a subsequent loss in glucose yield, (3) an order of magnitude improvement in the time required for pretreatment (minutes versus hours or days) can be realized using the catalyzed pretreatment approach, and (4) enzyme dosage can be reduced to less than 30 mg protein/g glucan and potentially further with only minor losses in glucose and xylose yields. In addition, we established that the reaction rate is improved in both catalyzed and uncatalyzed AHP pretreatment by increased solids concentrations.

**Conclusions:**

This work explored the relationship between reaction conditions impacting a catalyzed oxidative pretreatment of woody biomass and identified that significant decreases in the H_2_O_2_, catalyst, and enzyme loading on the biomass as well as decreases in the pretreatment time could be realized with only minor losses in the subsequent sugar released enzymatically. Together these changes would have positive implications for the economics of a process based on this pretreatment approach.

## Introduction

As the global demand for energy grows, the need for a sustainable fuel supply as a supplement or replacement for fossil fuels is becoming imperative [1]. Among possible technology options, the biochemical conversion of plant-derived sugars to biofuels has the potential to displace a substantial fraction of gasoline. This biochemical route involves the enzymatic hydrolysis of plant-derived polysaccharides to monomeric sugars, followed by fermentation of these sugars to biofuels such as ethanol. Starch from corn grain has been a major source of sugars for ethanol production in the U.S., but significant future growth of the corn ethanol industry is limited by the growing demand for both food and animal feed, as well as the recent achievement of maximum production limits on starch-based ethanol set by the Renewable Fuel Standard in the Energy Independence and Security Act of 2007 [2]. Thus, cellulosic biomass (*i.e.* plant cell wall material) is envisioned as an important feedstock for producing biofuel sustainably in the future as well as meeting renewable transportation fuel mandates.

Woody biomass is an especially attractive alternative to corn as a feedstock for biofuels. In particular, short-rotation woody bioenergy crops such as willow (*Salix* spp.) and hybrid poplar (*Populus* spp.) that are currently grown in temperate regions for combined heat and power bioenergy applications represent important feedstocks for liquid transportation fuels with agronomic and logistical advantages. Specifically, it has been shown that hybrid poplar can be grown on marginal agricultural lands with low energy and chemical input and produce biomass with high energy density at moderately high productivities [3,4], thereby providing significant motivation for developing effective and economic conversion technologies that can be coupled with woody feedstocks.

Due to the higher order structures in the plant cell wall, a chemical, thermal, or physical pretreatment step is necessary to facilitate the biochemical production of biofuels from plant cell wall polysaccharides. This need for pretreatment is primarily a consequence of cell wall lignin that limits cellulolytic enzyme accessibility to polysaccharides, with this cell wall recalcitrance to conversion especially problematic for the cell walls of woody plants. A wide range of pretreatments are known that differ in chemistry and mechanism but share the same outcome of increasing the accessibility of cell wall polysaccharides to cellulolytic enzymes [5]. Alkaline hydrogen peroxide (AHP) pretreatment is one such approach that has been studied since the 1980s [6-8]. AHP results in significant improvement in the enzymatic digestibility of commelinid monocots including corn stover and wheat straw [9,10] and can generate hydrolysates with more than 100 g/L of monomeric sugars that are fermentable without detoxification (manuscript in preparation). The mechanisms by which AHP pretreatment reduces recalcitrance include the fragmentation and solubilization of ester-linked xylan and lignin [11,12], as well as the oxidation, solubilization, and at high H_2_O_2_ loadings, mineralization of lignin [7]. While effective at increasing enzymatic digestibility, however, the majority of previous work employed high H_2_O_2_ loadings (*i.e.* >250 mg/g biomass) that would be economically challenging to implement industrially due to the high cost of H_2_O_2_[6,7], and it is well-established that oxidative delignification using H_2_O_2_ treatment under alkaline conditions requires high H_2_O_2_ loadings to realize significant lignin removal from wood pulps [13].

Relative to herbaceous monocots, woody biomass such as hybrid poplar presents special challenges for AHP because of its thicker cell walls, denser vascular structure, and higher lignin content [14,15]. As a result, the improvement in enzymatic digestibility after AHP pretreatment is limited [16,17], and this lack of efficacy on woody biomass is a ubiquitous challenge faced by many pretreatment methods [5,18,19]. Although a few methods including organosolv, dilute acid, and SPORL (a sulfite pretreatment combined with mechanical size reduction) have been reported to be effective pretreatments for hybrid poplar [20,21], all of these methods still have drawbacks such as a high consumption of chemicals and the generation of fermentation inhibitors [22]. As a result, there is great interest in identifying effective pretreatments for woody biomass.

In nature, plant cell walls can be biologically altered, catabolized, or degraded by plant pathogens and saprophytes including basidiomycetes [23], ascomycetes [24], and bacteria [25,26]. The strategies used by these organisms include the release of reactive oxygen species produced by redox-active metals and metalloenzymes [27] as well as the excretion of species-dependent combinations of lignin-modifying oxidoreductases [28-31], monooxygenases [32], and glycan-acting hydrolases, esterases, and lyases. Several abiotic catalytic oxidative treatments that mimic certain features of these successful biological approaches have recently been investigated as technologies for pulp bleaching or delignification [33,34] and the pretreatment of cellulosic biomass for the production of biofuels [35,36]. Leskelä and co-workers developed a pressurized O_2_-dependent strategy catalyzed by copper-diimine complexes that is effective in both pretreatment processes and pulp bleaching [36-38]. Another effective biomimetic approach reported by Lucas et al. uses oxidation by H_2_O_2_ catalyzed by manganese acetate to improve the hydrolysis yield from poplar sawdust [39].

We previously reported that AHP pretreatment catalyzed by copper(II) 2,2΄-bipyridine complexes (Cu(bpy)) can result in significant improvements in the enzymatic digestibility of a number of biomass feedstocks including switchgrass, silver birch, and most notably hybrid poplar [35]. The improved cellulose digestibility correlated with increased lignin removal as well as modifications to cellulose that included oxidative depolymerization, the introduction of carboxylate groups, and the solubilization and/or oxidative degradation of only up to 5% of the glucan in the original biomass. In the present manuscript we describe our investigation into the key parameters that impact the effectiveness of Cu(bpy)-catalyzed AHP pretreatment on hybrid poplar heartwood as quantified by glucose and xylose release during the subsequent enzymatic digestion of the pretreated biomass. Importantly, we report that the presence of catalytic amounts of Cu(bpy) during AHP pretreatment greatly improves process performance and decreases the required H_2_O_2_ loading, pretreatment time, enzyme loading, and hydrolysis time. Together, these reduced inputs result in significantly lower pretreatment costs and provide a compelling strategy for an improved pretreatment process for woody biomass.

## Results and discussion

The key factors that influence the effectiveness of Cu(bpy)-catalyzed AHP pretreatment are reactant concentrations, catalyst concentrations, and reaction times. These variables not only strongly influence the extent of sugar release, but also greatly affect the overall economics of the pretreatment process. For example, lower H_2_O_2_ and enzyme concentrations result in decreased raw material costs while increased reaction rates lower capital costs associated with reactor volume due to decreased residence time. The initial pH for uncatalyzed AHP is 11.5 (the approximate p*K*_a_ of H_2_O_2_), thereby largely defining the required NaOH loading. Therefore, in this work we focused on H_2_O_2_, biomass, and Cu(bpy) loadings, and how these variables affect pretreatment times, the required enzyme loading, and subsequent sugar release during saccharification.

**Figure 1:**
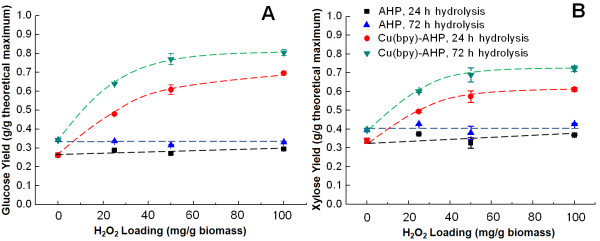
**Effect of H**_**2**_**O**_**2 **_**loading during pretreatment on enzymatic hydrolysis yields.** Results for **(A)** glucose and **(B)** xylose demonstrate that yields approach their saturation values near an H_2_O_2_ loading of 50 mg/g of biomass for Cu(bpy)-catalyzed AHP pretreatment. Pretreatment was performed for 24 h at 10% (w/v) solids with catalyzed pretreatment employing 2.0 mM catalyst concentration.

To identify a potential lower limit for H_2_O_2_ during Cu(bpy)-catalyzed AHP, the effect of H_2_O_2_ loading on the subsequent enzymatic glucose and xylose yield of pretreated hybrid poplar was tested (Figure 1). These results demonstrate that while there is only minimal improvement in glucose and xylose yields with increasing H_2_O_2_ loadings for uncatalyzed AHP, the presence of catalytic amounts of Cu(bpy) results in monomeric glucose yields of more than 80% and monomeric xylose yields of more than 70% at the highest H_2_O_2_ loading (100 mg/g biomass) after 72 h of hydrolysis. Importantly, these results demonstrate that the H_2_O_2_ loading can be halved (from 100 to 50 mg/g biomass) with less than a 4% decrease in the 72 h glucose and xylose yields. Additionally, the trend predicts that the H_2_O_2_ loading could be further decreased to as low as 35 mg/g biomass (comparable to loadings used in commercial pulp bleaching sequences [40,41]) and still result in more than 70% glucose yields for 72 h of hydrolysis. Considering that the cost of H_2_O_2_ would likely be one of the primary contributions to the raw materials costs, (along with biomass feedstock, enzyme, and catalyst cost) this 50-65% decrease in the H_2_O_2_ loading is substantial. For instance, based on a H_2_O_2_ cost of $1000/ton, this 65% reduction in peroxide loading would decrease the H_2_O_2_ cost from $0.305/kg of total sugar generated to only $0.081/kg.

**Figure 2:**
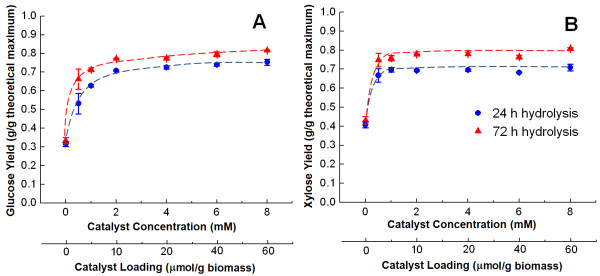
**Effect of catalyst concentration during Cu(bpy)-AHP pretreatment on the enzymatic hydrolysis yields.** Results for **(A)** glucose and **(B)** xylose, demonstrate that yields approach their saturation values near a Cu(bpy) concentration of 2 mM (or 10 μmol/g biomass). Pretreatment was performed for 24 h at a solids concentration of 20% (w/v) and a 100 mg/g H_2_O_2_ loading on the biomass.

The concentration of the Cu(bpy) catalyst utilized during pretreatment is another variable that can be optimized. This water-soluble metal complex has many advantages including its ease of synthesis from CuSO_4_ and 2,2'-bipyridine [42] and the fact that it is small enough to diffuse into nanoscale pores within plant cell walls to perform catalysis *in situ*. Reducing Cu(bpy) loadings would be advantageous because this would reduce input costs, alleviate potential copper inhibition to the fermentation microorganism, and diminish environmental concerns about the fate of the catalyst in process water treatment streams. To this end, we tested the effect of catalyst loading on the enzymatic digestibility of pretreated hybrid poplar (Figure 2). Our results demonstrate that after 24 h pretreatment at 20% solids loading, the glucose and xylose yields both essentially saturate at a Cu(bpy) concentration of 2.0 mM (corresponding to a catalyst loading of 10 μmol/g biomass) regardless of the hydrolysis time. In addition, the catalyst concentration can be further halved to 1.0 mM (5.0 μmol/g biomass) with only a 10% loss in the 72 h glucose yield (Figure 2A) and essentially no loss in the xylose yield (Figure 2B).

**Figure 3:**
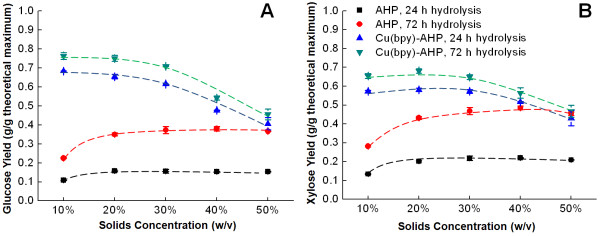
**Effect of solids concentrations during pretreatment on enzymatic hydrolysis yields.** The data for **(A)** glucose and **(B)** xylose, demonstrate inverse trends for Cu(bpy)-AHP versus uncatalyzed AHP. Pretreatment was performed for 24 h at 100 mg H_2_O_2_ per gram of biomass with catalyzed pretreatment employing 2.0 mM catalyst concentrations.

Importantly, the loading of catalyst on the basis of biomass can be decreased still further by increasing the solids concentrations during pretreatment while keeping the catalyst concentration constant (Table 1, Figure 3). Performing pretreatment and hydrolysis at high solids concentrations with no subsequent washing imparts a number of process benefits, including a decrease of process water usage, a decrease in required reactor volumes, and an increase in sugar titers from hydrolysis and subsequent ethanol titers from fermentation. Intriguingly, uncatalyzed AHP pretreatment shows a noticeable increase in glucan and xylan digestibilities as the hybrid poplar solids are increased from 10% to 20% (w/v), with further modest increases continuing even up to 50% (w/v) solids concentration. The catalyzed AHP pretreatment shows a different trend in that the maximum enzymatic digestibility of hybrid poplar is achieved for solids concentrations in the range of 10% to 20% (w/v) solids with pretreatment efficacy decreasing above 30% solids (w/v) concentration. We suspect that at higher solids concentrations (>20% w/v), the efficacy of the catalyzed pretreatment may be affected by limited mass transfer due to the lack of free water [43], decreased selectivity of H_2_O_2_ for the biomass versus disproportionation due to the change in reactant concentrations, and/or the decrease in catalyst loading on the biomass (decreasing to 4.0 μmol/g biomass).

**Table 1:** Chemical inputs used for pretreatment of 500 mg biomass at different solids concentrations

**Insoluble solids concentration (w/v)**	**Liquid volume (mL)**	**Cu catalyst**	**H**_**2**_**O**_**2 **_**loading on biomass (mg/g)**	**NaOH loading on biomass (mg/g)**
		**loading on biomass (μmol/g)**^**a**^		
10%	5.00	20	100	108
20%	2.50	10	100	108
30%	1.67	6.67	100	108
40%	1.25	5	100	108
50%	1.00	4	100	108

^a^In all cases the catalyst concentration is 2.0 mM.

**Figure 4:**
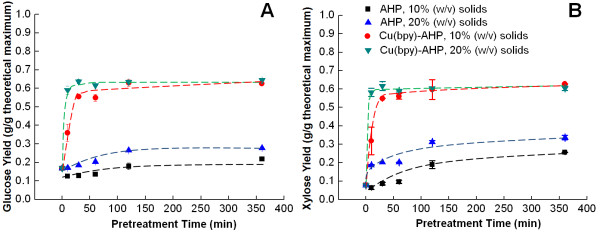
**Effect of pretreatment time and solids concentration on enzymatic hydrolysis yields.** The pretreatment kinetics showing enzymatic **(A)** glucose and **(B)** xylose release for catalyzed pretreatments were performed with 2.0 mM catalyst concentrations and 100 mg H_2_O_2_ per gram of biomass, and the hydrolysis was performed for 24 h.

Pretreatment reaction kinetics is important for the economics of a process since reactor volume and hence capital equipment requirement is proportional to the residence time of the reactor. An advantage of Cu(bpy)-catalyzed AHP pretreatment is that the pretreatment time is significantly shorter than for uncatalyzed pretreatment. In fact, the enzymatic glucan yield of pretreated poplar rapidly increases to approach a near maximum value within only 10–30 min pretreatment time at 10% solids (w/v) concentrations, while increasing the solids to 20% (w/v) results in achieving the maximum value in less than 10 min (Figure 4A). Comparable increases in the xylan yields can also be achieved within the same short period of time (Figure 4B). Conversely, uncatalyzed AHP pretreatment results in considerably lower yield improvements and requires significantly longer pretreatment time for maximum efficacy.

**Figure 5:**
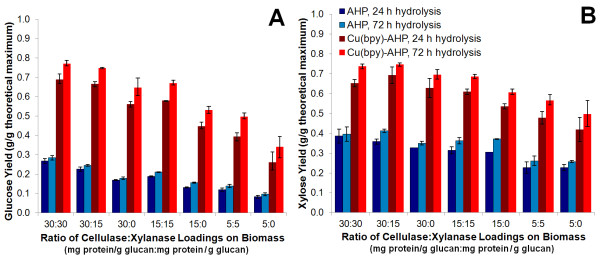
**Effect of enzyme loading and xylanase supplementation on enzymatic hydrolysis yields.** The results for **(A)** glucose and **(B)** xylose compare AHP and Cu(bpy)-catalyzed AHP pretreatment performed for 24 h with 100 mg H_2_O_2_ per gram of biomass, 10% (w/v) solids concentration, and a Cu(bpy) concentration of 2.0 mM for the catalyzed reaction.

In addition to employing enzymatic hydrolysis as a screen for determining the differences in pretreatment conditions, we also investigated both enzyme loading and xylanase supplementation for their impact on glucan and xylan yields. While an extensive optimization of enzyme cocktail was not performed, several enzyme loadings and cellulase:xylanase ratios were tested, with the results presented in Figure 5. This shows that the improvement in sugar yield between catalyzed and uncatalyzed pretreatment is significant for all enzyme loadings tested, but the greatest absolute difference is at the higher enzyme loadings. Furthermore, it can be observed that substantially less enzyme is needed to achieve higher digestibilities (*i.e.* less mass enzyme protein per mass sugar generated) using Cu(bpy)-catalyzed AHP treated poplar relative to the AHP pretreated material. Another observation is that the xylanase supplementation provides improvement in both the glucose and xylose yields with the synergy between xylanases and cellulases increased at limiting enzyme loadings (Figure 5). This indicates that, like other xylan-retaining pretreatments, xylanase leveraging is possible [44], although the optimal ratios cannot be established from this data. While this was not a complete enzyme cocktail optimization, these results indicate that for the given pretreatment conditions, glucan and xylan yields nearly saturate at their maximum achievable levels with respect to enzyme loading. Additionally, the enzyme dosage can be decreased by at least a 50% total enzyme loading of 30 mg protein/g glucan (at a 15:15 cellulase:xylanase ratio) with only minor losses in glucose and xylose yields. This relatively high enzyme loading could likely be decreased further if a full optimization were performed. This decrease is important considering that enzyme costs are anticipated to be one of largest contributions to cellulosic biofuels costs [45].

**Figure 6:**
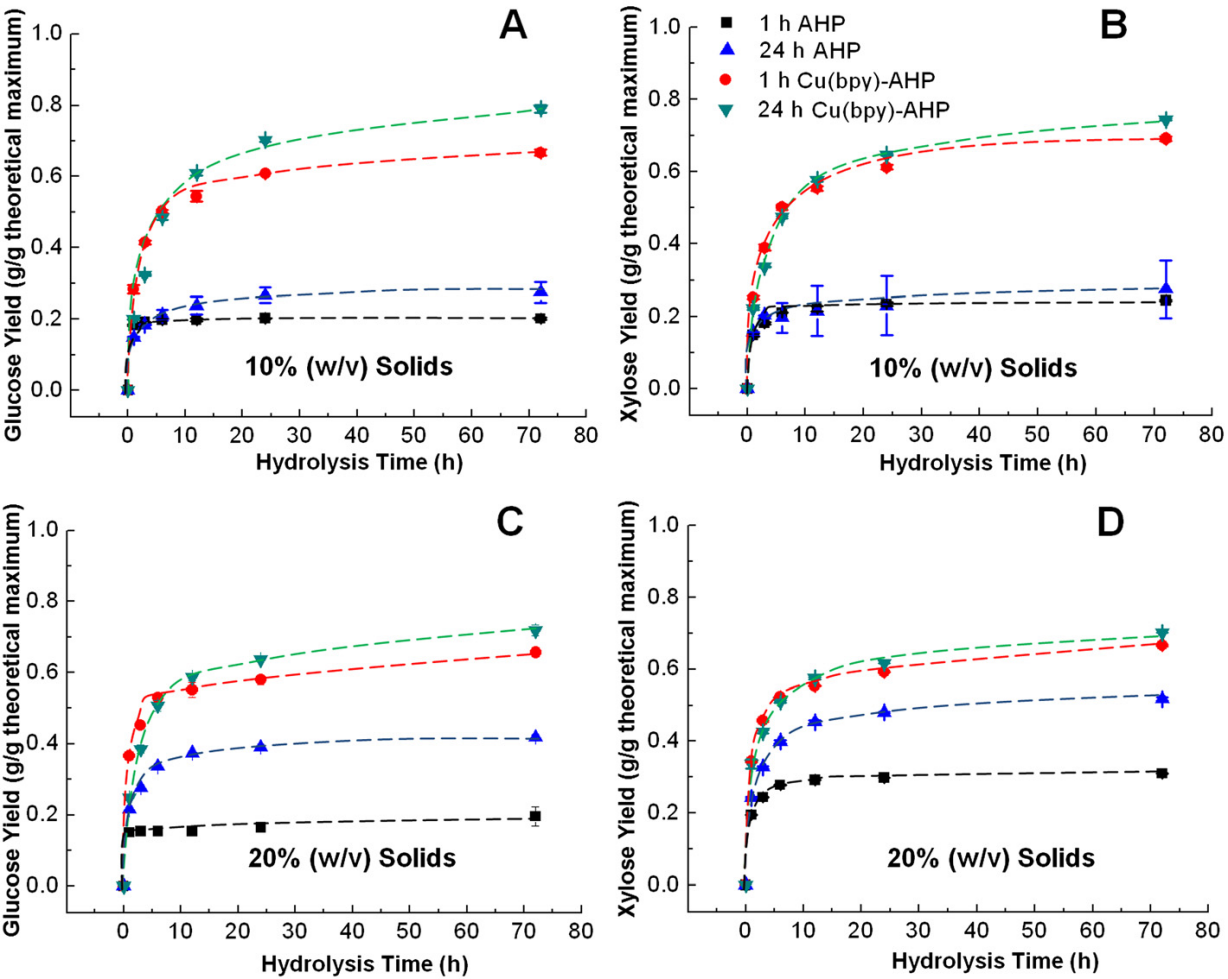
**Enzymatic hydrolysis kinetics for uncatalyzed and Cu(bpy)-catalyzed AHP.** The data show yields of glucose and **(A** and **C)** and xylose **(B** and **D)** and the solids concentrations (w/v) during pretreatment are 10% **(A** and **B)** and 20% **(C** and **D)**. Pretreatment was performed for 24 h with 100 mg H_2_O_2_ per gram of biomass at a catalyst concentration of 2.0 mM for Cu(bpy)-catalyzed AHP pretreatment.

The kinetics of the enzymatic hydrolysis for catalyzed and uncatalyzed pretreatment during pretreatment was also investigated (Figure 6), highlighting a number of important outcomes of the pretreatments. As demonstrated above, both the rate and extent of enzymatic hydrolysis are significantly improved following Cu(bpy)-catalyzed AHP treatment relative to uncatalyzed treatment. After 3 hours of hydrolysis, the enzymatic yields of glucose in Cu(bpy)-catalyzed AHP pretreated poplar is approximately two-fold higher than that in hybrid poplar after uncatalyzed AHP pretreatment, and this ratio increases even further with longer hydrolysis time. Another key finding is that while longer pretreatment times result in higher monomeric glucose yields for both catalyzed and uncatalyzed AHP pretreatment, the majority of the glucose yield improvement by pretreatment takes place within the first 30 minutes. Additionally, the differences in sugar yield between 1 h and 24 h pretreatment times nearly disappear at 20% solids, in agreement with previous results (Figure 4).

For both catalyzed and uncatalyzed AHP pretreatment, the xylose hydrolysis yields are highly correlated with the glucose hydrolysis yields (Figures 1, 2, 3, 4, 5 and 6). In fact, there is an almost perfect linear correlation (R-squared greater than 0.99) between glucose and xylose release for most conditions of Cu(bpy)-catalyzed AHP (Additional file 1: Figure S1A) with a slope of 1.5, meaning that every 15% increase in glucose yield corresponds to a 10% increase in xylose yield. This can be contrasted to uncatalyzed AHP pretreatment of hybrid poplar which results in lower yields of both glucose and xylose (less than 35% and 50% respectively), a weaker correlation (R-squared values ranging from 0.70 to greater than 0.99 for individual data sets), and slopes in the range of 0.60 to 0.85 (Additional file 1: Figure S1B). We speculate that these differences may have implications for both the mechanisms of pretreatment and its impact on the cell wall structure.

In general terms, improvements in enzymatic yields can be understood as cell wall alterations that provide hydrolytic enzymes greater accessibility to the cell wall matrix polysaccharides, and these differences in glucose versus xylose released may be interpreted as differences in how the pretreatments alter the polysaccharide accessibility to enzymes. Specifically, enzyme accessibility for cellulose hydrolysis is known to be dependent on xylan removal in conjunction with lignin removal or relocalization in diverse woody and herbaceous angiosperms. This relationship between xylan removal and cellulose hydrolysis has been shown for both dilute acid [46] and AHP [11] pretreatments, as well as during enzymatic hydrolysis whereby increased xylanase supplementation is well-known to increase the glucose yields for biomass where a significant fraction of the xylan is retained during pretreatment [44]. This is reasonable considering that xylan is believed to sheath the surface of cellulose microfibrils and modulate its interactions with other cellulose microfibrils as well as other cell wall matrix macromolecules [47]. We speculate that the uncatalyzed AHP pretreatment is only capable of removing easily accessible or easily extractable xylan and that only minor glucose hydrolysis yield improvements can be realized in this regime as the bulk of the cellulose may still be embedded within the lignified cell wall. The increase in the slope of the linear correlation in the high glucan digestibility regime (corresponding to pretreatment with Cu(bpy)-catalyzed AHP) can be interpreted as representing an enhanced total polysaccharide accessibility due to the action of pretreatment, whereby the increased accessibility of xylan and its hydrolysis results in an increase in cellulose hydrolysis. As shown in our previous work [35], this enhancement in polysaccharide accessibility during Cu(bpy)-catalyzed AHP pretreatment is possibly a consequence of improved lignin removal. Moreover, the depolymerization of lignocellulose macromolecules potentially disrupts cell wall structure, increases the area accessible to enzymes, and facilitates mass transfer during enzymatic hydrolysis.

## Conclusions

In this work we characterized Cu(bpy)-catalyzed AHP pretreatment conditions that resulted in significantly increased enzymatic saccharification yields for hybrid poplar heartwood. Relative to our previously reported results from an initial screening of catalysts and biomass feedstocks [35], here we describe a number of important improvements in this catalyzed pretreatment of woody biomass that will enable more efficient use of process inputs during pretreatment and enzymatic hydrolysis. These include significant decreases in the loading of H_2_O_2_ on biomass, catalyst concentrations, enzyme loadings, and pretreatment duration as well as an increase in solids concentrations. Importantly, it was found that the H_2_O_2_ loadings on biomass could be decreased to as low as 35–50 mg/g without significant losses in sugar yield by enzymatic hydrolysis, bringing the oxidant loadings down into the range that may be employed in commercial pulp bleaching sequences. Future investigations on Cu(bpy)-catalzyed AHP pretreatment will focus on enhancing our mechanistic understanding of the catalyzed oxidation, characterizing the changes to cell wall polymers and solubilized organics, and improving process integration with fermentation.

## Methods

### Chemical composition analysis of hybrid poplar heartwood

Heartwood from 18-year old hybrid poplar (*Populus nigra* var. *charkoviensis* x *caudina* cv. NE-19) grown at the University of Wisconsin Arlington Agricultural Research Station was hammermilled to pass through a 5 mm screen (Circ-U-Flow model 18-7-300, Schutte-Buffalo Hammermill, LLC). The initial composition of structural carbohydrates and acid-insoluble lignin (Klason lignin) were determined using the NREL two-stage acidolysis method [48] with modifications as described elsewhere [35].

### Catalytic AHP pretreatment

Copper(II) 2,2΄-bipyridine complexes were prepared *in situ* in an aqueous stock solution containing 15.6 g/L CuSO_4_·5H_2_O (EMD Chemicals, Billerica, MA) and 31.2 g/L 2,2΄-bipyridine (Sigma-Aldrich, St. Louis, MO) to yield a molar ligand to metal (L:M) ratio of 5:1. Hybrid poplar (0.500 g dry basis; 3.0% moisture) was pretreated in a total of 5.0 mL aqueous solution containing 2.0 mM Cu catalyst, 20.0 g/L H_2_O_2_, and 21.6 g/L NaOH for pretreatment at 10% solids. For pretreatment at different solids concentrations, a different volume of aqueous solution with the 2.0 mM Cu catalyst was used, while the doses of H_2_O_2_ and NaOH on the basis of biomass were the same (Table 1). After vortex mixing the reactants with the biomass, the slurry was incubated with orbital shaking at 180 rpm at 30°C [35].

### Enzymatic hydrolysis

After pretreatment, 20 μL of 72% (w/w) H_2_SO_4_ and 0.5 mL of 1 M citric acid buffer (pH 4.8) were added to the pretreated slurry to adjust the pH to 5.0, a level suitable for enzymatic hydrolysis. Next, 40 μL of 10 mM tetracycline (Sigma-Aldrich) stock solution was added to inhibit microbial growth, followed by addition of the enzyme cocktail consisting of Cellic CTec2 and HTec2 (Novozymes A/S, Bagsværd, DK) at a loading of 30 mg protein/g glucan each on the untreated biomass unless otherwise noted. The total protein contents of enzyme cocktails used in determining enzyme loadings on biomass were quantified using the Bradford Assay (Sigma–Aldrich). The total volume was adjusted to 10 mL by the addition of deionized water, and the samples were incubated at 50°C with orbital shaking at 180 rpm. Following enzymatic hydrolysis, the solid and liquid phases were separated by centrifugation, and the amount of glucose and xylose released into the aqueous phase was quantified by HPLC (Agilent 1100 Series equipped with an Aminex HPX-87H column operating at 65°C, a mobile phase of 0.05 M H_2_SO_4_, a flow rate of 0.6 mL/min, and detection by refractive index) [35]. The yield of glucose and xylose released was defined as the amount of solubilized monosaccharide divided by the total sugar content of the biomass prior to pretreatment as determined by chemical composition analysis [48]. While xylan is solubilized during pretreatment, no monomeric sugars were detected in the pretreatment liquor. Error bars in figures represent the data range between biological replicates.

## Abbreviations

AHP: Alkaline hydrogen peroxide; Cu(bpy): Copper(II)-2,2΄-bipyridine complexes; HPLC: High performance liquid chromatography.

## Competing interests

The authors declare that they have no competing interests.

## Authors’ contributions

DBH, ELH and ZL conceived the work, ZL, CHC, DBH, and ELH wrote the manuscript, CHC performed the pretreatment, composition analysis and enzymatic digestibility analysis. All authors provided input and corrections to the manuscript. All authors read and approved the final manuscript.

## Supplementary Material

Additional file 1: Figure S1 Replotting data from Figures 1 and 6 demonstrating different trends in glucose and xylose yield between catalyzed (A) and uncatalyzed (B) AHP pretreatment. Click here for file
