# Supplementary figures and images for: Rapid and effective oxidative pretreatment of woody biomass at mild reaction conditions and low oxidant loadings
Source: Biotechnol Biofuels. 2013 Aug 26;6:119. doi: 10.1186/1754-6834-6-119 (PMC3765420; doi:10.1186/1754-6834-6-119)

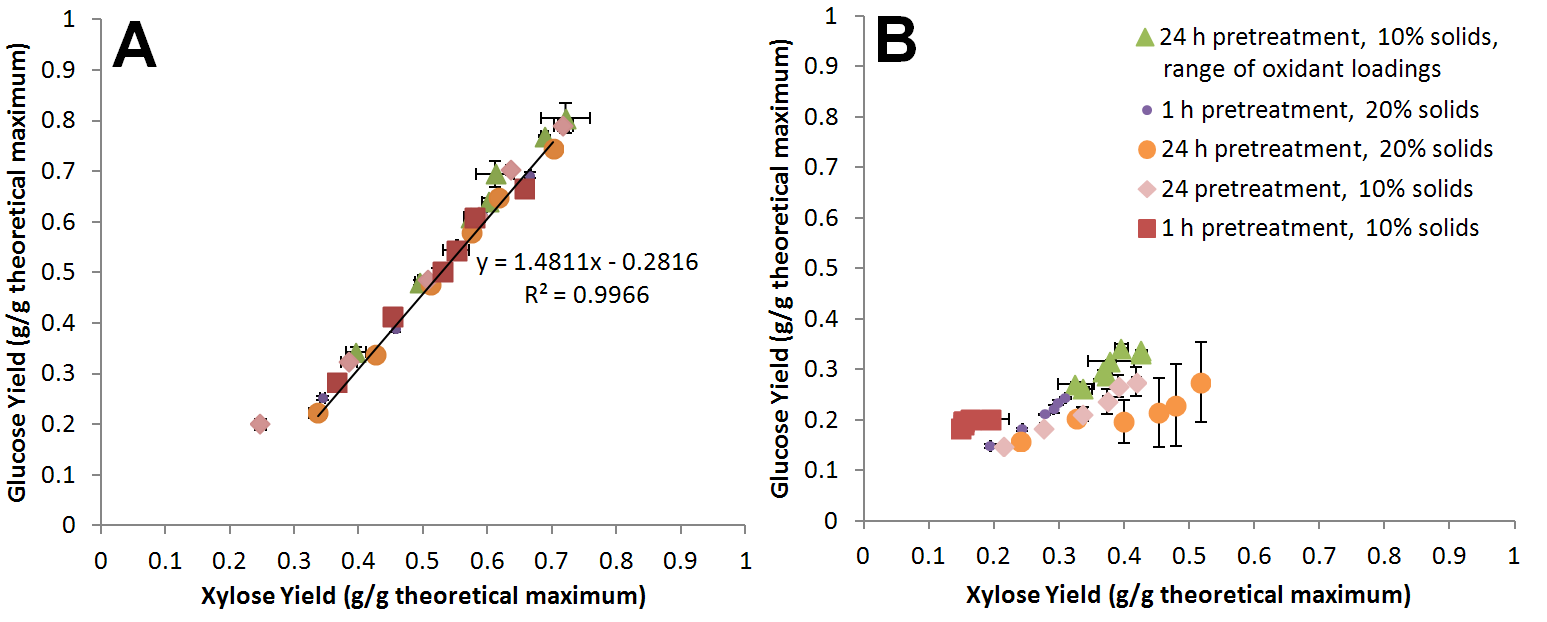

Supplement: Additional file 1: Figure S1 — Replotting data from Figures 1 and 6 demonstrating different trends in glucose and xylose yield between catalyzed (A) and uncatalyzed (B) AHP pretreatment. [file 1754-6834-6-119-S1.tiff]
